# Marine protected areas do not prevent marine heatwave-induced fish community structure changes in a temperate transition zone

**DOI:** 10.1038/s41598-020-77885-3

**Published:** 2020-12-03

**Authors:** R. M. Freedman, J. A. Brown, C. Caldow, J. E. Caselle

**Affiliations:** 1 NOAA Channel Islands National Marine Sanctuary, University of California, Santa Barbara, Ocean Science Education Building 514 MC 6155, Santa Barbara, CA USA; 2 Ecology Evolution and Marine Biology Department, University of California Santa Barbara, Santa Barbara, CA USA; 3 ECOS Consulting, LLC, Lafayette, CA USA; 4 Marine Science Institute, University of California Santa Barbara, Santa Barbara, CA USA

**Keywords:** Ecology, Climate-change ecology, Community ecology, Conservation biology, Marine biology

## Abstract

Acute climate events like marine heatwaves have the potential to temporarily or permanently alter community structure with effects on biodiversity and ecosystem services. We aimed to quantify the magnitude and consistency of climate driven community shifts inside and outside Marine Protected Areas before and after a marine heatwave using a kelp forest fish community dataset in southern California, USA. Abundance, biomass, diversity and recruitment of warm-water affinity species during the marine heatwave were significantly greater compared with prior years yet cool-water affinity species did not show commensurate declines. Fish communities inside MPAs were not buffered from these community shifts. This result is likely because the particular species most responsible for the community response to environmental drivers, were not fisheries targets. Resource managers working to preserve biodiversity in a changing climate will need to consider additional management tools and strategies in combination with protected areas to mitigate the effect of warming on marine communities.

## Introduction

Marine heatwaves are severe, acute thermal events that elevate water temperatures and significantly impact marine ecosystems^1–5^. Marine heatwaves—defined as events of > 5 days where water temperatures exceed the 90th percentile of the 30-year historical baseline^6^—are predicted to increase in frequency and intensity globally^2,7,8^. Studies show marine heatwaves can rapidly push ecosystems into new states, with shifts happening within a few months in some systems^2,3,5^. As marine heatwaves become more common world-wide due to global climate change^7^, a better understanding of how marine communities respond to these events and what management tools might mitigate them, will become critical to effective ocean resource management.

While species are generally predicted to shift their ranges in response to warming conditions^9^, individual communities will have unique responses depending on sensitivities to local environmental conditions^10–14^. There is evidence that some species will benefit from marine heatwaves (i.e. increase in biomass or frequency) while others are negatively impacted^2–4,12,14–18^. As local community structure is altered by local extinction and re-colonization in response to shifting climate, it is likely that ecosystem function and services will be altered as well. Understanding the magnitude and consistency of these changes will be key to determining how to best manage resources in the face of both punctuated and sustained climate shifts^4,5,11,15^.

Marine Protected Areas (MPAs) are a global management tool that are frequently promoted as potentially useful in buffering local communities from climate impacts^19^. Spatial management, specifically the designation of protected area networks, can support resistance and resilience of marine communities^3,20–24^ and there is some evidence that these benefits could apply to climate pressures, including heatwaves^24^. Some managers assume existing MPAs can mitigate impacts to whole ecosystems and their structures in the face of acute and long-term climate impacts. There is evidence that MPAs can help single species recover from heatwaves^22^ and may offer diversity stability in the face of climate drivers^3^. However, the extent to which MPAs can mitigate large community structural changes in the face of marine heatwaves is unknown at present^24^.

**Figure 1:**
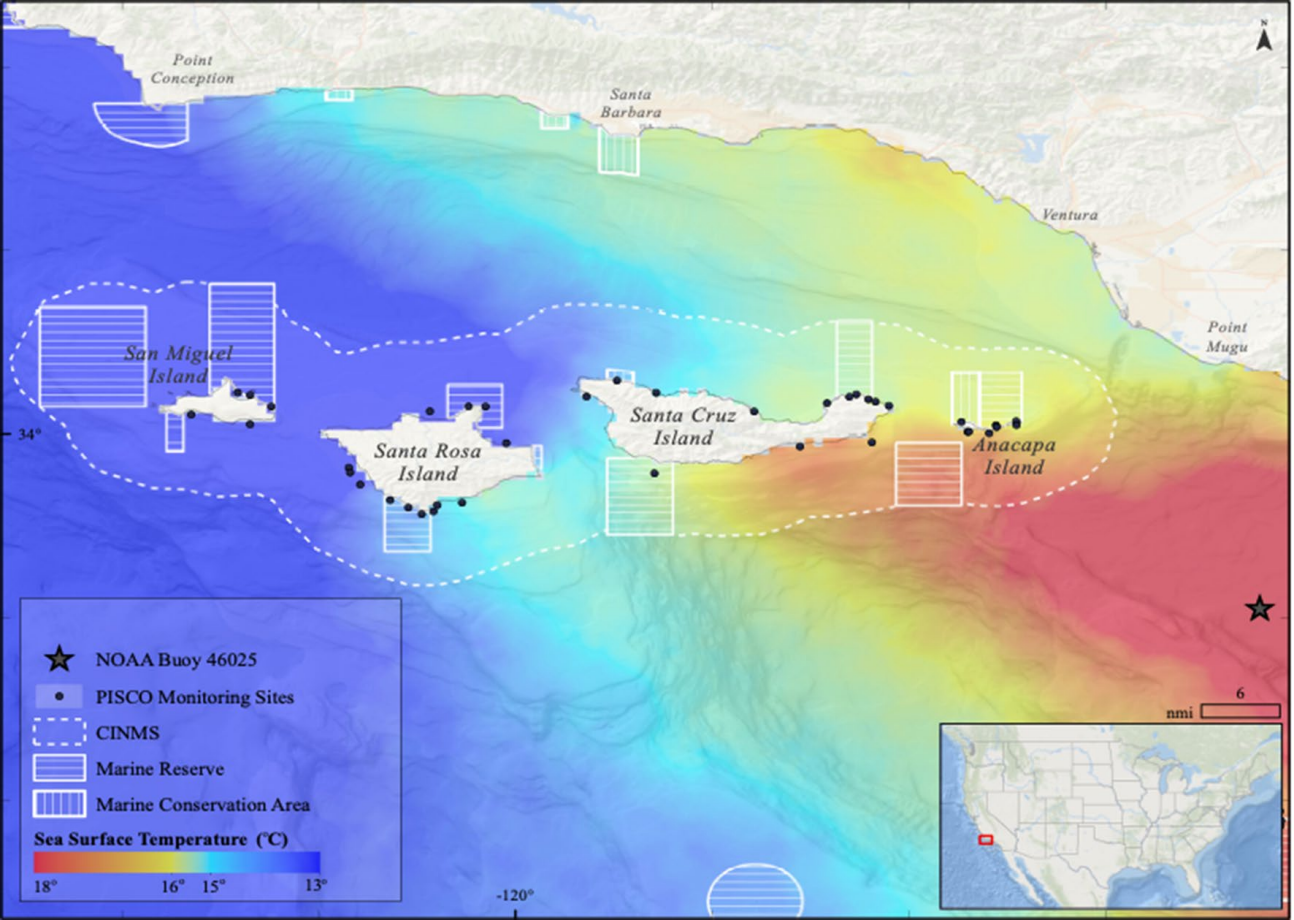
The Channel Islands National Marine Sanctuary (CINMS) and the Marine Protected Area (MPA) network are shown in a composite 2009 sea surface temperature grid, a thermally typical year. Typically, the islands span a strong east–west gradient in sea surface temperature due to their location at the confluence of the California Current and the California countercurrent. This variable environment was replaced with a more uniformly warm SST regime during the 2014–2016 marine heatwave. PISCO long-term kelp forest monitoring sites are shown as black dots. Map was created using ArcGIS 10.7.1 (https://desktop.arcgis.com/).

Here we aimed to address two goals: quantifying the magnitude and consistency of changes in response to marine heatwaves and determining MPA’s ability to mitigate changes in community structure of kelp forest fishes in a well-known biogeographic transition zone (Fig. 1). We first measured the response of the kelp forest fish community to a marine heatwave (October 2014–June 2016). The marine heatwave caused persistent temperature anomalies (up to 6.2 °C) off the US west coast^25–27^ and has been shown to have caused ecological impacts to kelp forest communities^28^. We selected the Channel Islands National Marine Sanctuary (CINMS) in the Santa Barbara Channel, CA, USA as our study site as it is a region at the confluence of the cooler California Current and warmer California Counter Current, is marked by a steep west to east thermal gradient, and has long-term kelp forest monitoring data spanning several climatic periods^20,29,30^. By conducting this work in a marine transition zone many species are at their range limits and will be sensitive to small changes in environmental conditions. Using the thermal classification scheme developed by Freedman^31^ and 17 years of kelp forest monitoring data in the region (2001–2017; Partnership for the Interdisciplinary Studies of Oceans [PISCO]), we tested whether abundance, biomass, recruitment and diversity of warm-water and cool-water affiliated fishes responded to the marine heatwave and whether responses differed between the thermal groups. We used Bayesian Highest Density Interval and Region of Practical Equivalence tests (HDI + ROPE) to assess annual similarity amongst warm-water and cool-water species groups^32,33^. We then asked whether fish communities inside MPAs are more or less susceptible to changes in community structure during marine heatwaves using a long-standing no-take MPA network in our study area. Using data collected just prior to (2013), during (2014–2015) and just after (2016) the marine heatwave, we tested whether warm-water and cool-water species groups differed in their density responses inside and outside of MPAs. To further interpret MPA efficacy, we subdivided warm-water and cool-water species groups as targeted (i.e., fished) and non-targeted (i.e., not fished) and then asked if densities of these groups responded differently to the marine heatwave.

### Quantifying heatwave impacts to the fish community

**Figure 2:**
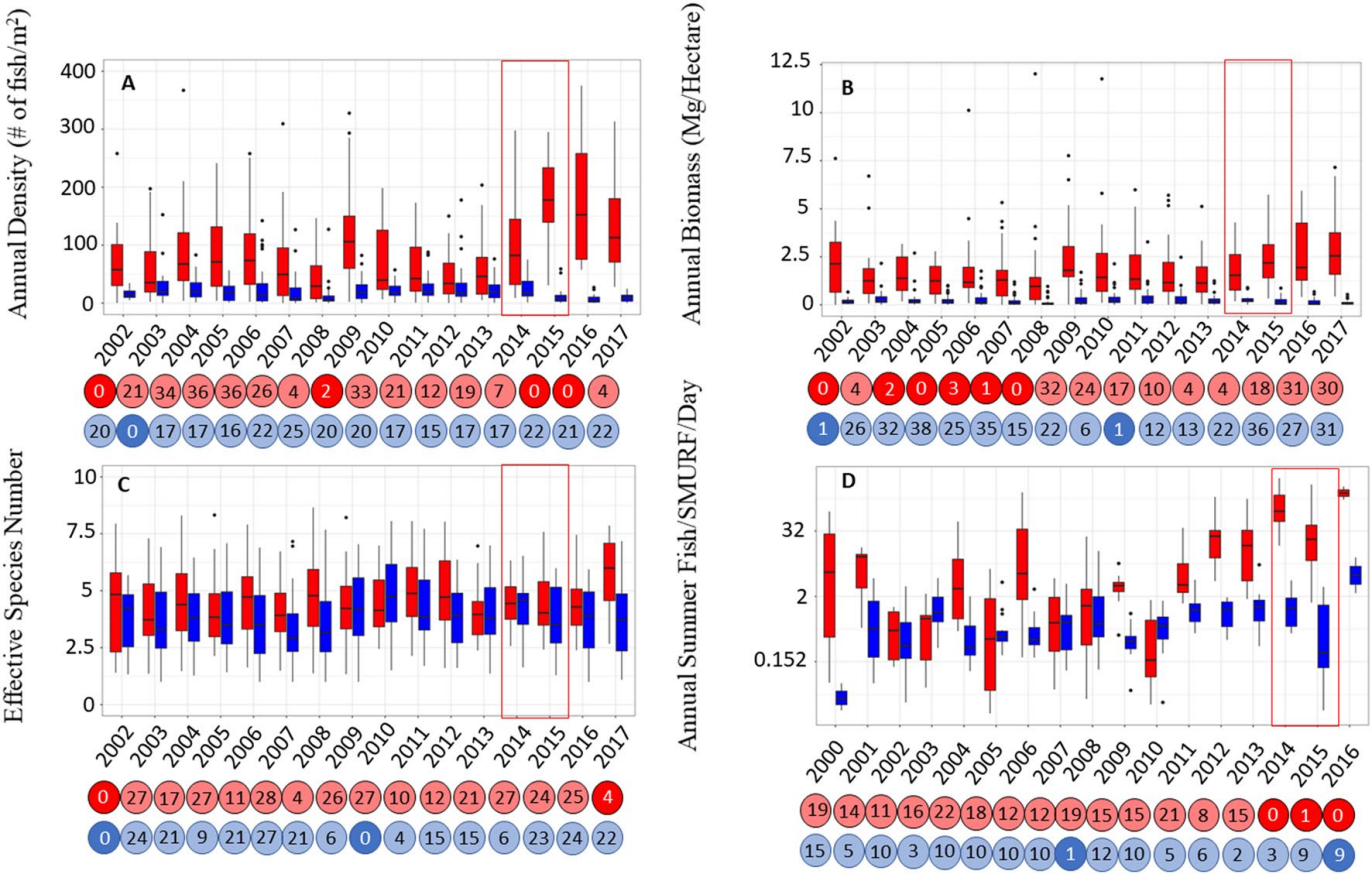
Time series of annual density (**A**), biomass density (**B**), effective species number (**C**), and recruitment (**D**) are shown for warm-water species (red) and cool-water species (blue). Region of Practical Equivalence and Highest Density Interval (ROPE + HDI) tests results are denoted by circles below panels. Darker shaded circles identify years that fall outside the ROPE and are significantly different than others in the time series. Numbers inside the circle are the approximate percentage of data that fall within the ROPE within that year. Significant increases in density, effective species and recruitment of the warm-water species group was observed during the marine heatwave period (as denoted by the red box).

The marine heatwave had a significant impact on the structure of local fish communities, and this effect was dependent on the thermal affinity as well as the targeted or non-targeted status of the fish species. In fish density and recruitment, the warm-water fishes responded positively and rapidly to the marine heatwave while the cool-water species were generally less responsive. The density of warm-water species significantly increased during (2015) and remained high after the marine heatwave (2016, Fig. 2A). In 2017, warm-water species density came back into the ROPE which may be indicative of beginning recovery but it also had the highest median compared with any year outside of the marine heatwave. Warm-water species recruitment also spiked rapidly and significantly (i.e. beyond the ROPE—see “Methods”) at the onset of the marine heatwave (2014) and remained high until 2016 (Fig. 2D). Diversity within the warm-water species group lagged in its response (Fig. 2C), only rising above the ROPE in 2017, over a full year after the marine heatwave subsided. Detection of diversity responses may lag periods of anomalous temperature conditions due to sampling methodology missing rare species, time needed for adult migration or multiple years of strong larval recruitment.

The cool-water species group appeared less responsive overall to warm-water conditions. Although cool-water species did display non-significant short-term decreases in density, biomass density, and diversity over the timeframe of the marine heatwave (Fig. 2A–C), they were insignificant in comparison to historical variability. Cool-water species recruitment responded significantly to the end of the marine heatwave with a large spike of recruitment in 2016 after multiple years of warmer water conditions (Fig. 2D). The lack of significant responses for this group suggests that cool-water species in the region might be less susceptible to rapid climate drivers. In our system, the cool-water species are typically larger and longer-lived species of the genus Sebastes (Rockfishes) and are characterized by slower life histories^12,29,34^. Previous work on rockfishes has linked their slow response to management action to their slow life history traits^34^. Other studies have found that fishery productivity responses, both positive and negative, to warming climate are faster for fishes with shorter life histories and these species also shift ranges more rapidly in response to climate^14,15^. Because life histories can determine species-specific climate responses, cool-water species may be less responsive to acute climate events in the California Current; however chronic exposure to warm conditions may still pose a threat to cool-water fishes^35–37^.

Of the four demographic measures that we assessed, biomass was less responsive to the marine heatwave in the Channel Islands than numerical density. The increase in biomass for the warm-water species group after the onset of the marine heatwave was not statistically significant (Fig. 2B). Warm-water species biomass was below the ROPE in 2002 and from 2004 to 2008; potentially due to a lag in biomass gains following MPA implementation in 2003. Other work in the Channel Islands region has found biomass increases to be spatially variable after MPA implementation with fishes around the warmer eastern islands displaying larger and more rapid changes in biomass compared to the cooler western islands^29^. Because MPA implementation increased biomass of warm-water species throughout the time-series, the biomass variability across the dataset may mask the effects of the marine heatwave. Cool-water species biomass also displayed some evidence of a decline in response to the marine heatwave, but the declines were not severe enough to fall below the ROPE.

The fish community also responded to less severe, “routine” climatic drivers like El Niño and La Niña; however, responses were less extreme and did not persist long after the event (~ 1 year). Cool-water species diversity in 2010 (Fig. 2C), biomass in 2011 (Fig. 2B) and recruitment in 2008 (Fig. 2D) increased beyond the ROPE during cold La Niña conditions. Warm-water species densities were also higher than the ROPE in 2009 during a moderate El Niño event. Response signals to routine climatic events for warm-water species are not as prevalent across all monitoring data; possibly due to their extreme responses to the marine heatwave event masking the ability of the HDI + ROPE tests to detect milder responses to less severe climatic drivers like El Niño.

### Assessing MPA effectiveness to mitigate heatwave induced community structure shifts

**Figure 3:**
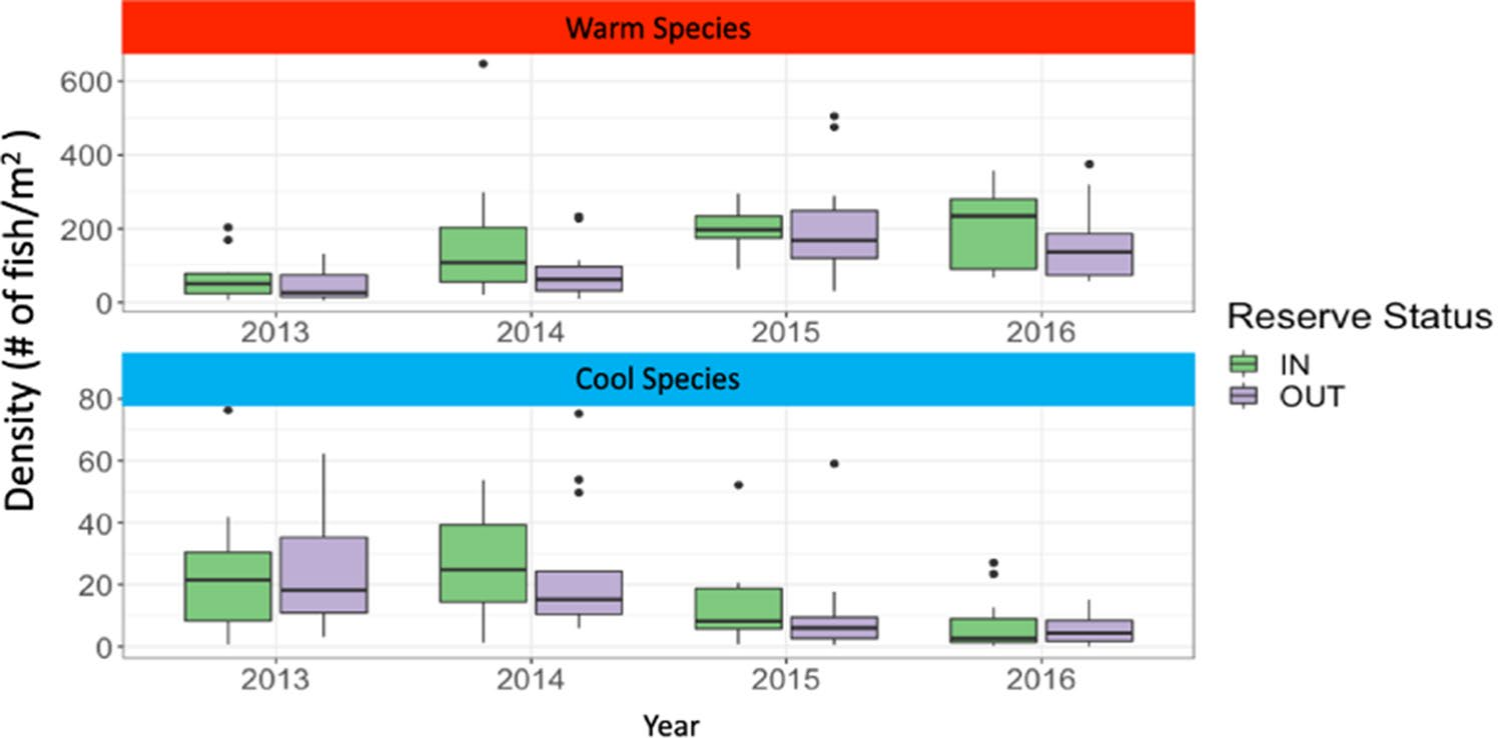
Density of warm-water species (top panel) and cool-water species (bottom panel) inside (green) and outside (purple) MPAs in the years before (2013) during (2014–2015) and after (2016) the marine heatwave. Warm-water species density increased during the marine heatwave while cool-water species density decreased regardless of MPA protection. Linear mixed models found that Year was the only significant factor (warm-water species: ANOVA, X^2^ = 36.11, df = 3, p < .0001; cool-water species ANOVA, X^2^ = 26.23, df = 3, p < .001) while MPA status (warm-water species: ANOVA, X^2^ = 0.79, df = 1, p = 0.37; cool-water species ANOVA, X^2^ = 0.07, df = 1, p = 0.79) and the interaction between year and MPA status (warm-water species: ANOVA, X^2^ = 4.41, df = 3, p = 0.22; cool-water species ANOVA, X^2^ = 0.31, df = 3, p = 0.96) were non-significant.

Just as species with different traits may respond differentially to climate drivers^38–40^, they also react uniquely to different conservation measures^3,22,29,40–42^. Using linear mixed models, we found that MPAs did not mitigate the observed community shifts in numerical density resulting from the marine heatwave (Fig. 3). The density of warm-water species increased significantly over time (Fig. 3, Supplemental Table 1) and the density of the cool-water species group decreased over time (Fig. 3, Supplemental Table 1) but there was no effect of MPA status on these trends. That is, density of warm-water species increased similarly both inside and outside MPAs, while cool-water species showed similar declines inside and outside. Prior work has shown that single species recovery after an acute climate event can be enhanced within an MPA^22^, but our work suggests that MPAs do not appear to dampen the magnitude of community changes for either cool or warm-water species groups in our system. Additionally, in our case, ecosystem recovery did not appear to be rapid; densities inside and outside MPAs remained altered and overall warm-water species abundance did not drop to pre-heatwave values by 2016. It is important to note that using an acute event may not be indicative of long-term change, but it appears that MPAs are unlikely to act as a lone solution to mitigate the whole community structure shifts due to climate change^43^.

**Figure 4:**
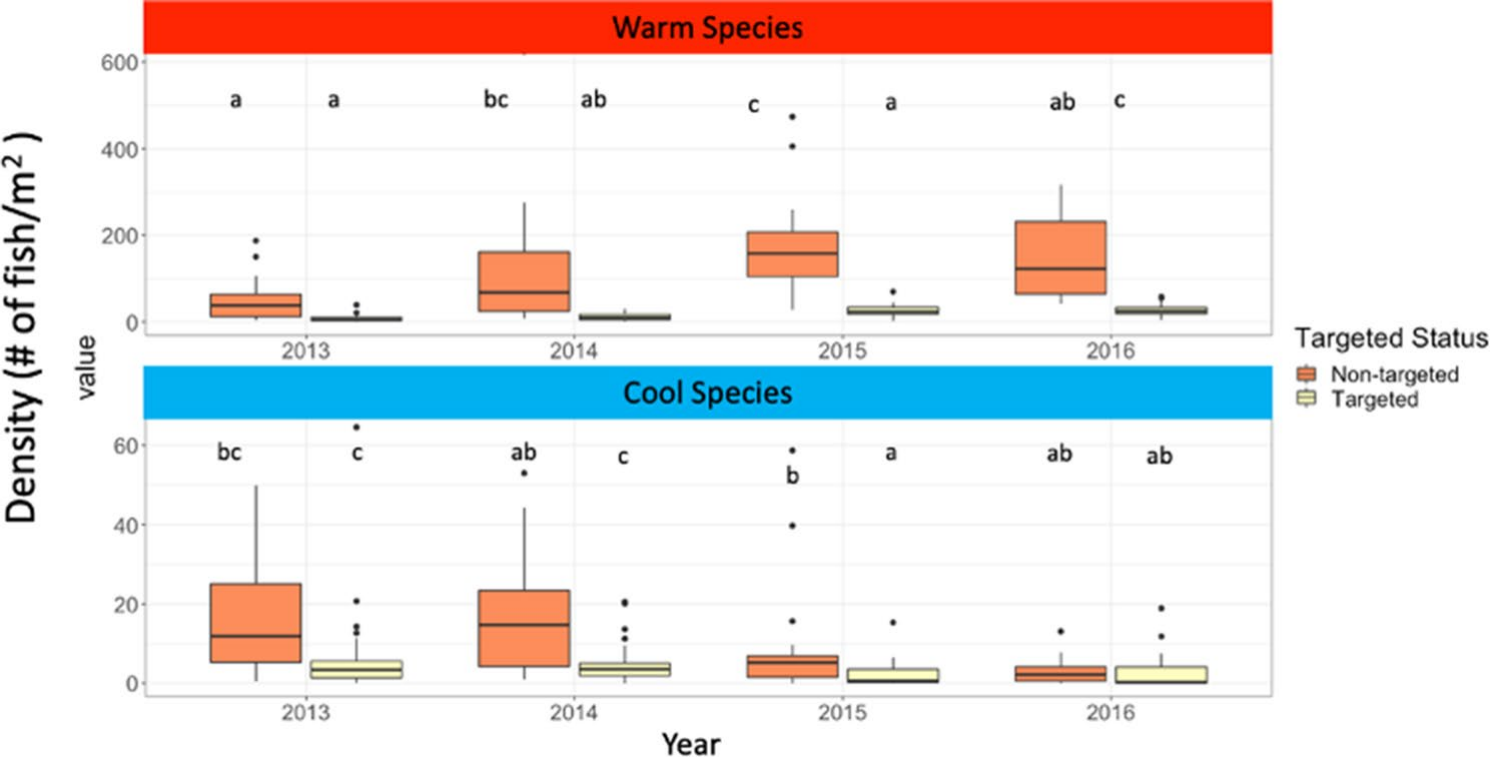
Density of warm-water species (top panel) and cool-water species (bottom panel) for both targeted (yellow) and non-targeted (orange) species in the years before (2013) during (2014–2015) and after (2016) the marine heatwave. Non-targeted species responded more strongly to the marine heatwave with warm-water species densities rising while cool-water species densities decreased. Linear mixed models found that effects of targeted status (warm-water species: ANOVA, X^2^ = 118.69, df = 1, p < .001; cool-water species ANOVA, X^2^ = 23.43, df = 1, p < .001), year (ANOVA, X^2^ = 30.55, df = 3, p < .001; cool-water species ANOVA, X^2^ = 26.20, df = 1, p < .001) and the interactions between the two (ANOVA, X^2^ = 19.68, df = 3, p < .001; cool-water species ANOVA, X^2^ = 9.89, df = 3, p = .019) were all significant. Post-hoc comparisons show that density of non-targeted species changed during the marine heatwave with warm-water species (top panel) increasing and cool-water species (bottom panel) decreasing.

The reason MPAs did not appear to mitigate marine heatwave impacts on community structure in our study may be that non-targeted species (i.e. fish species not targeted in fisheries) responded significantly to the marine heatwave while targeted species only displayed non-significant, muted responses. Thus, the very species we expect to most benefit from cessation of fishing, were not the species most effected by the heatwave. According to linear mixed models, targeted status, year and the interactions between the two (Supplemental Table 2) were important in driving the communities’ response to the marine heatwave. Targeted species in this, and many marine systems are typically larger, longer-lived, and higher in the food web^44,45^, which may make them more resilient to climate events of this magnitude^14^. In our classification scheme, targeted cool species outnumber the non-targeted species while the inverse is true for warm-water species. This means that the strong response by non-targeted warm species is buoyed by the fact that there are more species in those categories then in others^31^. Marine heatwaves with higher temperature maximums or that persist for longer than a few years may be required to trigger targeted species to respond. Because targeted species already have lower abundance than non-targeted species in our system (Fig. 4), detecting changes in abundance in relation to climate stressors may be a challenge. The complex synergies of management strategies, fishing and climate are still poorly understood. Further research will be needed to disentangle these effects, but our work shows that MPAs are likely not the only tools managers should be utilizing if they wish to mitigate the effects of marine heatwaves.

## Conclusion

This study showed that the structure of kelp forest fish communities was responsive to acute climate forcing and was altered after exposure to a marine heatwave. The potential mechanisms producing this shift in community structure include mortality, adult movement (especially to cooler, deeper waters beyond the sampling depth) and recruitment; for example, recruitment of warm-water species increased after the heatwave began. It is likely that each of these mechanisms contributed to the overall changes observed in the kelp forest fish community, however, the exact contribution is likely to vary across the diversity of species in this dataset. Additional data would be needed to fully elucidate the role of each underlying mechanisms within this diverse community. Rather than use a whole-community thermal index^5,16,46^, we showed that there were unique responses for groups with different thermal affinities. With density and recruitment being the most responsive, managers may want to focus on these metrics as rapid and sensitive indicators to change while biomass and density should be used to understand lagging or persistent community shifts. With climate warming predicted to continue and accelerate, an improved understanding of trait-specific responses will become important^31,46^; especially if families of species or ecological niches are lost to a degree that hampers ecosystem function. Species classification techniques, in this case thermal tolerances, can help resource managers better track particular resource species or species groups responses to distinct environmental drivers (e.g., temperature, pH, hypoxia, drought) and test if management actions are effectively mitigating climate stressors. We found that a scientifically designed and very well studied network of MPAs in our system^47^, did not mitigate the rapid responses in density of the two thermal groupings of kelp forest fishes. Additional data will be needed to assess if MPAs allow for more rapid recovery after heatwave events but managers hoping to preserve whole ecosystems may need to explore other avenues to locally mitigate effects of climate change in conjunction with MPA measures, such as restoration or stock enhancements. As the majority of community structure change was driven by non-targeted species, adjusting fishing pressure (by MPAs or other methods) may not be a viable avenue to address community level effects of acute temperature events unless fishing pressure is reallocated to previously non-targeted warm-water species. Other work has shown that a number of fisheries stocks see increases in Maximum Sustainable Yield from warming^14^ and managers may need to consider increasing utilization of these specific stocks. Whatever management decisions are made, resource managers need to have flexible and dynamic management measures as marine heatwaves will likely continue into the future and act as consistent disturbance events in temperate oceans worldwide.

## Methods

### Site

The Channel Islands National Marine Sanctuary (Fig. 1) is located in an established marine thermal transition zone between the cooler California Current and the warmer California Countercurrent. Four islands with rocky reef habitat are situated offshore, separated from the mainland by a deep basin, the Santa Barbara Channel. The two water bodies mix in approximately the middle of the Santa Barbara Channel and water temperatures can differ as much as 10 °C across the channel. Fishing activity occurs heavily in the region with three major fishing ports supporting commercial and recreational fisheries that target a variety of species.

A variety of marine spatial management protections and jurisdictions co-occur in the channel including Channel Islands National Marine Sanctuary, Channel Islands National Park, two California State Parks and a series of no-take and limited-take MPAs. The MPAs, which are the only spatial management measure that restricts fishing, were established in 2003 in state waters (< 3 nm) and expanded into federal waters (> 3 nm) in 2007. Long-term monitoring studies of kelp forest fish communities in the Santa Barbara Channel detected rapid recovery of abundance and biomass—after MPA implementation, but this pattern was observed primarily at the eastern islands located in typically warmer waters^29^.

The Santa Barbara Channel has been subjected to a series of periodic climatic drivers with the potential to impact the ecosystem including El Niño Southern Oscillation Index, Pacific Decadal Oscillation, a marine heatwave beginning in 2014 and variable upwelling strength (Supplemental Figure 1). There was a weak El Niño from 2006 to 2007 which was followed by a weak La Niña in 2008–2009. This was followed by a stronger ENSO with a moderate El Niño in 2009–2010 that raised water temperatures and dampened upwelling. This was followed by a strong La Niña in 2010–2011 that reversed conditions. In 2014, a marine heatwave increased water temperature and those conditions persisted until June 2016. We consider data collected during the summer of 2016 to be post-marine heatwave as the large majority data collected was after June 2016.

### Classification

Kelp forest fish species were classified as warm-water or cool-water based on each species’ biogeographic distribution and abundance patterns relative to Point Conception (warm-water species centered to the south and cool-water species centered to the north of this Point) as described in Freedman^31^. Three types of quantitative data were used to assign thermal classification: densities from in situ surveys, museum/aquarium collection events, and the geographic range midpoints from literature sources. In addition to quantitative data, an expert opinion poll was used to further classify species. Data from each of the four information sources were combined to create a single composite thermal classification for each fish species. Equal weight was given to each data type and fish were considered to be warm-water or cool-water based on the dominant classification (i.e., the one to which it was most often assigned). If no clear classification was apparent, the species was considered eurythermal. Species classified as eurythermal constituted a small proportion of species observed in monitoring data and were not used in further quantitative analysis.

### Subtidal diver surveys

Fish species densities and biomass were generated from SCUBA surveys conducted by the Partnership for Interdisciplinary Studies of Coastal Oceans (PISCO; full methodology can be found online at http://www.piscoweb.org). The data used in this study were collected from 59 sites that were sampled annually from June to October; however, not all sites were surveyed in all years. The number of sampling sites increased in 2003 to capture the effects of the newly established MPAs. At each study site, divers conducted 8 to 12 transects that were 30 × 2 ×  2  m at each of three levels in the water column: benthic, midwater and kelp canopy (when canopy was present at a site). Transect locations were selected through a stratified random design with multiple non-permanent transects located in fixed strata (e.g. outer, middle, and inner rocky reef). On each transect, a single SCUBA diver counted and estimated the total length in centimeters for each fish, excluding small cryptic fishes. Values (fish/m^2^) were averaged across all transects and then summed by site annually for each thermal group (i.e., warm-water and cool-water). In order to estimate biomass, fish lengths were converted to weights based on allometric relationships in published and web-based sources (http://www.fishbase.org). When length–weight relationships did not exist, parameters from similar-bodied congeners were used. Shannon’s diversity was calculated by site annually for warm-water and cool-water species assemblages and transformed into effective species numbers, also known as Hill Numbers^48^, by taking the exponential function of Shannon’s diversity. Hill numbers were used to provide a comparable metric between years.

### Recruitment

Recruitment of larval fishes to the kelp forest was measured using artificial larval fish collectors (Standardized Monitoring Units for Recruitment of Fishes—SMURFs)^49^. Rates of settlement to SMURFs provide quantifiable measures of larval delivery independent of availability and quality of nearby settlement habitat. At each of seven sites at the Channel Islands, three replicate SMURFs were sampled bi-weekly and individual recruit fish were identified to the lowest taxonomic level possible. Further methods for PISCO SMURF collections can be found in Hamilton et al.^40^. Recruitment to SMURFs was first calculated as the number of fish per SMURF per day in order to standardize for slight variations in sample frequency. Recruitment (Fish per SMURF per day) was then summed by site and year for warm-water and cool-water species separately.

### Bayesian highest density internals (HDI) and region of practical equivalence (ROPE)

In order to identify when a community shifted from a baseline reference, Bayesian Highest Density Internals (HDI) and Region of Practical Equivalence (ROPE) testing was conducted on density, biomass, effective species number and recruitment for warm-water and cool-water species groups separately using the ‘sjstats’ package in R^32,33,50^. A 95% HDI value was determined for each parameter (e.g., annual density, biomass, effective species number, recruitment) and compared to that parameter’s ROPE; ROPE sizes were determined by the variability of the full timeseries for each dataset. When a parameter’s HDI fell within the ROPE, all the most credible parameter values were practically equivalent to the accepted value (i.e., the fish community at that time point was practically equivalent to others in the timeseries). A parameter value was rejected when its 95% HDI falls entirely outside the ROPE, which means that all of the most credible parameter values in the time series were not practically equivalent to those of the rejected value (i.e., the fish community at that time point was different from others in the timeseries). This methodology also allows for undecided determinations, where HDI was neither completely within or outside the ROPE. HDI and ROPE testing was conducted on each data type for warm-water and cool-water species groups independently with site as a random effect.

### Assessing MPAs’ ability to mitigate effects of acute climate drivers

Using the marine heatwave as an in situ experiment, we truncated the subtidal survey data to the years immediately before (2013), during (2014–2015), and after (2016) the marine heatwave. To test if MPAs had an effect on fish density during the marine heatwave period, we used linear mixed models for warm-water and cool-water species independently with year, MPAs status (inside/outside an MPA) and the interaction between the two as fixed effects on density with site as a random effect. We used density as it was the rapidly responsive variable to the marine heatwave in the prior analysis. All three MPA types were included as species being discussed are not allowed to be taken in all MPA types. Using the R package “nlme”, linear mixed models were built stepwise and the best fitting models were compared with Akaike Information Criterion (AIC)^51^. To test if targeted status (i.e., if a fish species is targeted by fishing by either recreational or commercial interest) affected density changes during the marine heatwave period, we used linear mixed models for warm-water and cool-water species groups with year, targeted status and the interaction between the two as fixed effects on density with site as random effect. Linear mixed models were built stepwise and compared with AIC to determine models of best fit. ANOVAs were run on the results of each best fitting model to determine significant effects, using Tukey’s post-hoc comparison between groups when ANOVAs were significant (lsmeans package R)^52^.

## Supplementary information

Supplementary Information 1.

Supplementary Information 2.
