# Supplementary material for: Marine protected areas do not prevent marine heatwave-induced fish community structure changes in a temperate transition zone
Source: Sci Rep. 2020 Dec 3;10:21081. doi: 10.1038/s41598-020-77885-3 (PMC7712829; doi:10.1038/s41598-020-77885-3)
Supplement: Supplementary file 1 — Supplementary Information 1. [file 41598_2020_77885_MOESM1_ESM.docx]

**Marine Protected Areas Do Not Prevent Marine Heatwave-Induced Fish Community Structure Changes in a Temperate Transition Zone**

**Supplementary Tables**

R. Freedman*^1,2^, J. Brown^1,3^, C. Caldow^1^, and J.E. Caselle^4^

Intended Journal: Scientific Reports

1: NOAA Channel Islands National Marine Sanctuary,

2. Ecology Evolution and Marine Biology Department, University of California Santa Barbara

3: ECOS Consulting, LLC

4: Marine Science Institute, University of California Santa Barbara

* Denotes Corresponding Author

NOAA Channel Islands National Marine Sanctuary

University of California, Santa Barbara

Ocean Science Education Building 514 MC 6155

Phone: 847-287-6661

Email: [ryan.m.freedman@noaa.gov](mailto:ryan.m.freedman@noaa.gov)

*Keywords*: Fish community structure, Marine Heatwave, Marine Protected Area, tropicalization, biogeographic transition zone

**Supplemental Table 1. ANOVA results for the linear mixed models comparing the effects of MPA status, year and the interaction between the two on fish density.**

| Warm-water Species Model |  | *X*^2^ | Df | p-value |
| --- | --- | --- | --- | --- |
|  | *Year* | *36.11* | *3* | *<0.0001* |
|  | Reserve | 0.79 | 1 | 0.37 |
|  | Year:Reserve | 4.41 | 3 | 0.22 |
| Cool-water Species Model |  |  |  |  |
|  | *Year* | *26.23* | *3* | *0.001* |
|  | Reserve | 0.07 | 1 | 0.79 |
|  | Year:Reserve | 0.31 | 3 | 0.96 |

**Supplemental Table 2. ANOVA results for the linear mixed models comparing the effects of targeted Status, year and the interaction between the two on fish density.**

| Warm-water Species Model |  | *X*^2^ | Df | p-value |
| --- | --- | --- | --- | --- |
|  | *Year* | *30.55* | *3* | *<0.001* |
|  | *Targeted Status* | *118.69* | *1* | *<0.001* |
|  | *Year:Targeted* | *19.68* | *3* | *<0.001* |
| Cool-water Species Model |  |  |  |  |
|  | *Year* | *26.20* | *3* | *<0.001* |
|  | *Targeted Status* | *23.43* | *1* | *<0.001* |
|  | *Year:Targeted* | *9.89* | *3* | *0.019* |
