# Supplementary figures and images for: Marine protected areas do not prevent marine heatwave-induced fish community structure changes in a temperate transition zone
Source: Sci Rep. 2020 Dec 3;10:21081. doi: 10.1038/s41598-020-77885-3 (PMC7712829; doi:10.1038/s41598-020-77885-3)

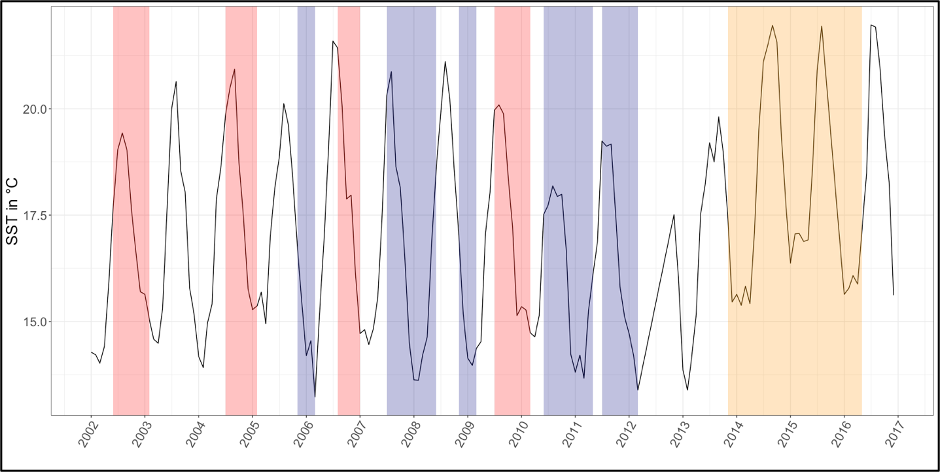

Supplement: Supplementary file 2 — Supplementary Information 2. [file 41598_2020_77885_MOESM2_ESM.png]
